# Removal of a Tumour from the Muscular Spiral Nerve

**Published:** 1830-01-01

**Authors:** 


### XVIII.

### NAVAL HOSPITAL OF ST. PETERSBURGH.

### REMOVAL OF A TUMOUR OF THE SPIRAL NERVE.*

This case is recorded by Harry Leeke

* Ed. Surg. Journ. No. CI. Oct. 1829.

Gibbs, M.D., in our respected northern contemporary. The patient was a sailor, æt. 42, admitted on the 24th December, 1828, with a tumour the size of a small hen's egg, about four inches above the right elbow-joint, just beneath the insertion of the deltoid. It was tolerably moveable, but connected by a cord both above and below, very painful when handled, the pain being lancinating and following the course of the branches of the radial nerve. He stated that fifteen years previously he received a blow with a handspike on the outside of the arm, and that he suffered for several months from pain and numbness stretching to the back of the hand and fingers. In the course of six months he first perceived a moveable subcutaneous swelling the size of a kidney bean, painful to the touch, and accompanied with lancinating pain in the direction of the nerve, which was worse during changes of weather or violent exertion. For twelve years and a half it remained stationary and had not increased until two years before his admission, when it did so gradually after long-continued hard labour. After proper antiphlogistic regimen, Dr. Gibbs proceeded to extirpate the disease on the 3d of January last.

"The arm being laid on a table, I made an incision of five inches, extending from the lower part of the deltoid to within an inch of the outer condyle of the *os brachii.* On dissecting back the integuments, a tumour of a bluish white colour was brought into view. I found it occupying the fossa, formed by the insertion of the *deltoid* muscle above, the *triceps extensor cubiti* behind, the origin of the *supinator radii longus* below, and the *brachialis externus* within. Under the outer portion of the latter muscle part of the tumour had embedded and attached itself, so that it became necessary to remove it. Having separated the tumour from its lateral attachments, I divided the nerve three quarters of an inch below it. The man instantly exclaimed that he had lost the power of raising his fingers, as well as the feeling in the outer part of the fore-arm and back of the hand. The dissection beneath was now easy, and I was happy to find that my fears of an adhesion to the periosteum were groundless. I lastly cut through the nerve at the like distance above the tumour, just as it emerges from beneath the bone. This was attended with pain, and as the artery accompanying the nerve bled freely, a ligature was applied. The vessel seemed dilated and to be the *arteria nutriens* of the tumour. The wound was brought together by strips of adhesive plaster, over which graduated compresses and a circular bandage were applied. The limb was placed on a pillow in a relaxed position, the fore-arm and hand enveloped in flannel, and a bottle of warm water ordered to be constantly applied to the fingers."

On the 4th there was so much constriction across the lower part of the sternum and præcordia, and pain shooting up the right side of the neck, that two pounds of blood were abstracted and an active purge administered. On the 5th a blister was applied, and on the 7th about half the wound was healed by the first intention, but there was acute pain at the extremity of the right thumb nail, and there was loss of sensibility in the parts supplied by the muscular spiral nerve. After a time electricity and stimulating frictions were employed, and on the 16th of April the patient was dismissed with returning sensation in the back of the hand, and a tolerably free use of the arm.

"The tumour on examination was found to consist externally of the thickened neurilema, extending from the nerve above the tumour as a capsule to the nerve below. This general tunic was of a lamellated structure, as viewed by the microscope, dense and inelastic, and of the colour of the *tunica albuginea* of the testis. Under this the nervous fasciculi appeared diverging and intersecting each other like net-work, to the thickness of half an inch. The rest of the mass consisted internally of a pulpy, striated, greenish-black matter, the striæ running from the circumference towards the centre, and surrounding minute interstitial cells filled with a similar medullary pulp or jelly. A

small portion of coagulated blood was very distinct on one side in the firmer substance of the tumour. The centre was occupied by half a tea-spoonful of highly fetid coffee-coloured sanies. Condensed cellular membrane surrounded the whole of the tumour, which at the inner and lower side was firmly adherent to that part of the *brachialis externus* removed with it during the operation. The tumour was an oval of two inches and a half in the long diameter, by nearly two inches across. This, with the portions of nerve divided above and below, brought the whole length of the extirpated part to four inches.

"*Observations.*—That the blow received on the arm fifteen years ago was the primary cause of the chronic inflammation established in the radial nerve, most probably first in its neurilematic theca or capsule, there is little doubt; but that it should have once commenced and then have lain dormant for the space of nearly thirteen years, is remarkable. This reproductive power I have had occasion to observe not unfrequently both in the mammæ and testes, arising either from a blow, the action of cold, or some peculiarity of the system at the time. The epididymis I have found to be the most speedily affected by this sudden change, particularly in dram-drinkers, and in those addicted to excess in venery. In these cases it appears to me that the sooner an operation is had recourse to, the better. The preparation of this rare and interesting tumour I have presented to the Hunterian Museum belonging to the Royal College of Surgeons of London."

The above is a very good example of those tumours connected with a nerve or its neurilema, which have been described by authors, and are occasionally met with in practice. We do not altogether share in Dr. Gibbs' surprise respecting the cessation of troublesome symptoms for the long interval of twelve years, as such temporary pauses are far from uncommon in the march of many organic diseases. Who, for instance, has not seen scirrhus occasionally lie dormant for a very considerable period of time, only to be kindled up at last into furious malignancy, and rapid disorganization?

